# Evaluation of introgressive hybridization among Cervidae in Japan's Kinki District via two novel genetic markers developed from public NGS data

**DOI:** 10.1002/ece3.5131

**Published:** 2019-04-26

**Authors:** Yuki Matsumoto, Toshihito Takagi, Ryosuke Koda, Akira Tanave, Asuka Yamashiro, Hidetoshi B. Tamate

**Affiliations:** ^1^ Department of Genetics, School of Life Sciences SOKENDAI Shizuoka Japan; ^2^ Mouse Genomics Resource Laboratory National Institute of Genetics Shizuoka Japan; ^3^ Graduate School of Science and Engineering Yamagata University Yamagata Japan; ^4^ Research Institute of Environment, Agriculture and Fisheries Neyagawa Japan; ^5^ Science and Technology, Graduate School of Technology, Industrial and Social Science Tokushima University Tokushima Japan; ^6^ Faculty of Science Yamagata University Yamagata Japan; ^7^ Present address: Research and Development Section Anicom Specialty Medical Institute Inc. Yokohama Japan

**Keywords:** *Cervus nippontaiouanus*, Formosan sika deer, introduced species, introgression, next‐generation sequencing, Okinoshima Island

## Abstract

Hybridization and backcrossing of native populations with introduced species can lead to introgression and genetic alteration. In this study, we evaluated introgression in 43 deer from a potential hybrid zone around Okinoshima Island, Kinki District, Japan. This region witnessed the migration of a hybrid population (cross between the Formosan sika deer [*Cervus nippon taiouanus*] and other deer species) that could potentially breed with the native Japanese sika deer (*C. n. centralis*). We used an existing genetic marker for the mitochondrial *cytochrome b* gene and two novel markers for nuclear DNA, developed using publicly available next‐generation sequencing data. We identified one mainland deer with a mitochondrial haplotype identical to that of the Formosan sika deer as well as nuclear heterozygous sequences identical to those of Formosan and Japanese sika deer. This suggests that the mainland deer is a hybrid offspring of the Okinoshima population and native deer. However, only Japanese sika deer sequences were found in the other 42 samples, indicating limited introgression. Nevertheless, hybridization pre‐ and postintroduction in the Okinoshima population could cause multispecies introgression among Japanese sika deer, negatively affecting genetic integrity. We developed a simple test based on polymerase chain reaction–restriction fragment length polymorphism to detect introgression in natural populations. Our method can accelerate genetic monitoring of Japanese sika deer in Kinki District. In conclusion, to prevent further introgression and maintain genetic integrity of Japanese sika deer, we recommend establishing fences around Okinoshima Island to limit migration, besides a continued genetic monitoring of the native deer.

## 1 INTRODUCTION

Invasive species hybridizing with related native species can result in the latter's extinction through introgression, eroding genetic integrity (Rhymer & Simberloff, 1996). In Cervidae, sympatric species easily hybridize and produce fertile offspring (Matsumoto, Ju, Yamashiro, & Yamashiro, 2015). One notable example is the introduction of sika deer (*Cervus nippon*) into Europe, North America, and New Zealand (McCullough, Kaji, & Takatsuki, 2009). In Scotland, native red deer (*C. elaphus*) populations are threatened (Abernethy, 1994) by repeated introduction of Japanese sika deer since the 19th century (Goodman, Barton, Swanson, Abernethy, & Pemberton, 1999), resulting in deer hybridization throughout the country (Smith et al., 2018). To conserve the native species, hybridization with the introduced species should be prevented through management methods such as isolation of the two populations.

The sika deer inhabiting Japan are highly divergent in terms of morphologic (Terada, Tatsuzawa, & Saitoh, 2012) and genetic features (Nagata et al., 1999). Morphologically, the Japanese sika deer are classified into six subspecies: *Cervus nippon yesoensis*, *C. n. centralis*, *C. n. nippon*, *C. n. mageshimae*, *C. n. yakushimae*, and *C. n. keramae*. Striking variation in body size and antler size are observed among subspecies (e.g., the typical body weight of *C. n. yesoensis* is 120 kg, whereas that of *C. n. keramae* is 30 kg) (Ohtaishi, 1986). Contrastingly, molecular genetic evidence for mitochondrial DNA indicated the existence of two major groups, namely the Northern and Southern Japan groups (Nagata et al., 1999). Although genetic evidence was not consistent with the morphology‐based classification, the latter is widely accepted at present (Nagata, 2009).

Formosan sika deer (*C. n. taiouanus*) is a subspecies native to Taiwan but now extinct in the wild (McCullough et al., 2009). In 1955, 10 individuals were introduced in Okinoshima Island, Wakayama Prefecture, Kinki District, Japan (Fukushima et al., 1983; Takatsuki, 1985) for tourism purposes (Tokida, 1998). The Okinoshima population exhibits considerable morphological variation, making identification of subspecies through morphological traits difficult (Matsumoto et al., 2015). Likely originated from a private deer farm in Taiwan, this population has identical mitochondrial *cytochrome b* and nuclear alpha‐lactalbumin sequences to those of Formosan sika deer, Formosan sambar (*C. unicolor swinhoei*), and red deer (*C. elaphus*) (Matsumoto et al., 2015).

**Figure 1:**
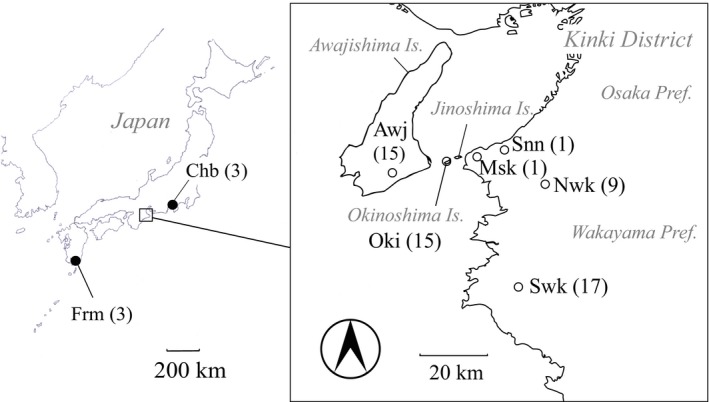
Sampling location of the control population and target population. Chb and Frm indicate the control samples of Japanese sika deer in Central Japan and Formosan sika deer from the Hirakawa Zoo (filled circles). Open circles indicate Okinoshima (Oki), the mainland Honshu (Misaki Town: Msk, Sennan City: Snn, North Wakayama: Nwk, and South Wakayama: Swk) and Awajishima (Awj) populations, respectively

During 2015–2016, one buck was caught in Sennan City and a doe was caught nearby, in Misaki Town; both locations are in the Honshu (mainland) portion of Kinki District and 4.2 km from Okinoshima Island (Figure 1). Deer were not known to be distributed around these Honshu areas previously. Sika deer can swim up to 12 km (Feldhamer, 1980), and individuals have been observed swimming around the island (Tatsuzawa, 2000). Therefore, there is a distinct possibility that the captured deer originated from the Okinoshima population and could hybridize with the native Japanese sika deer *(C. n. centralis*) on Honshu. Another population at risk of breeding with the Okinoshima deer is the Japanese sika on Awajishima Island, located 4 km west of Okinoshima Island.

Through genetic data, hybridization between species can be reliably determined (Goodman et al., 1999), and the success rate increases with the increase in the number of genetic markers (Dupuis, Roe, & Sperling, 2012). Mitochondrial DNA is a commonly used marker, but because they can only infer maternal inheritance, nuclear genetic markers such as single‐nucleotide polymorphisms (SNPs) should also be incorporated in hybridization analyses to identify paternal origin. However, only one nuclear marker that can distinguish between Formosan and Japanese sika deer is currently available (Matsumoto et al., 2015). Hence, more nuclear markers with moderate polymorphism in Cervidae must be identified.

**Figure 2:**
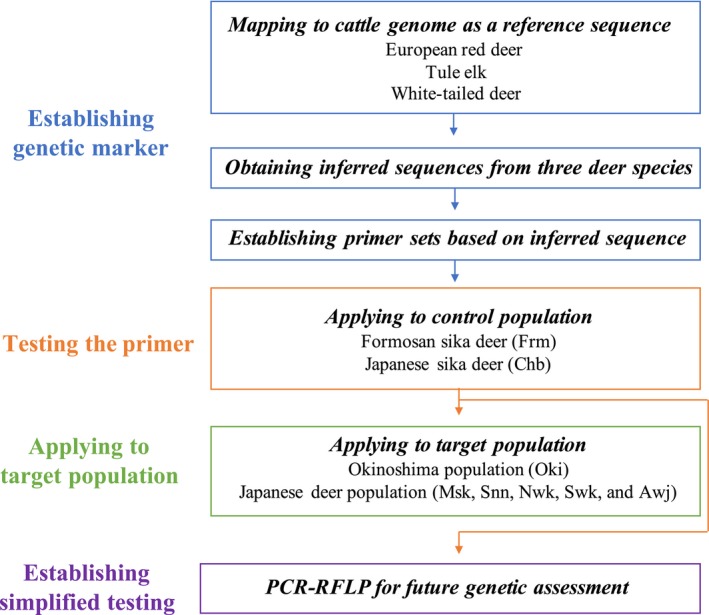
Schematic representation of the experimental procedures performed in this study

With the development of next‐generation sequencing (NGS), a large genetic dataset is now freely available in public databases such as the NCBI Sequence Read Archive (SRA) (Leinonen, Sugawara, & Shumway, 2011; https://www.ncbi.nlm.nih.gov/sra). In the present study, we developed two novel nuclear DNA markers using the NGS dataset (Figure 2) and included an existing mitochondrial marker to evaluate the genetic status of deer populations in Kinki District. Specifically, our aim was to determine the phylogeny and extent of introgression among Okinoshima deer and Japanese sika deer on Honshu and Awajishima Islands. We also established a new genotyping protocol using the polymerase chain reaction–restriction fragment length polymorphism (PCR‐RFLP) analysis as an alternative to direct sequencing (Amjadi, Varidi, Marashi, Javadmanesh, & Ghovvati, 2012; Meyer, Höfelein, Lüthy, & Candrian, 1995; Partis et al., 2000). Advantages of PCR‐RFLP include lower cost and higher speed (Amjadi et al., 2012; Meyer et al., 1995; Partis et al., 2000).

## 2 MATERIALS AND METHODS

### 2.1 DNA samples

As a control to evaluate novel DNA markers, blood and muscle samples were collected from three Formosan sika deer (that either died of natural causes or were subjected to medical treatment) at the Hirakawa Zoo in Japan (Frm). Three other control samples (Chb) were obtained from legally hunted Japanese sika deer in Shizuoka and Yamanashi Prefectures (Central Japan). These individuals were selected as they are unlikely to have experienced crossbreeding with the Okinoshima population, given that the sampling sites are 350 km apart.

We collected muscle or antler samples from deer representing three mainland populations (Figure 1): Misaki and Sennan from Osaka Prefecture (*n* = 1 each), North Wakayama Prefecture (*n* = 9), South Wakayama Prefecture (*n* = 17), and two island populations (Okinoshima Island, *n* = 14; Awajishima Island, *n* = 15). Non‐Okinoshima deer samples were collected from legally hunted individuals. Okinoshima deer samples were obtained from deer that died of natural causes between 2012 and 2016.

### 2.2 Establishing genetic markers based on NGS data

Novel genetic markers were developed using high‐quality and highly variable (HQHV) regions with elevated SNP count per 500 bp. A length of 500 bp was selected because one run of Sanger sequencing is sufficient for diagnosing DNA sequences of this size (Luo, Tsementzi, Kyrpides, Read, & Konstantinidis, 2012). To obtain the HQHV region, we first searched the NCBI SRA (https://trace.ncbi.nlm.nih.gov/Traces/sra/sra.cgi?view=software) for NGS raw reads of one European red deer (*C. e. hippelaphus*) (Bana et al., 2018), four tule elk (*C. canadensis nannodes*) (Sacks, Lounsberry, Kalani, Meredith, & Langner, 2016), and one white‐tailed deer (*Odocoileus virginianus*) (Seabury et al., 2011) (Table 1). Using the better‐established cattle (*Bos taurus*) reference genome (UMD3.1) from the Bovine Genome Database (http://bovinegenome.org/), we extracted the HQHV region in the deer genome. The raw read data (sra files) were converted to the fastaq format using SRA Toolkit v2.8.2 (Leinonen et al., 2011). These fastq files were then mapped to the bovine reference genome using the BWA‐MEM function in bwa 0.7.15 (Li & Durbin, 2009). Subsequently, SNPs were called and extracted from high‐quality mapped regions using samtools v1.3.1 (Li, 2011) and vcftools v0.1.15 (Danecek et al., 2011). Inferred sequences in the three deer species were constructed from SNPs with EMBOSS cons (http://www.bioinformatics.nl/cgi-bin/emboss/cons). Coverage depth was calculated for each site using R and bash scripts written by the authors. Regions were removed if the absolute value of mean coverage ± 0.1 was exceeded; this criterion avoids the negative influence of duplicated genomic regions. Regions with <1% N (undetermined nucleotide site) and heterozygous sites were also excluded to avoid low mapping quality. Regions with >1 SNP per 100 bp were considered highly variable and extracted. Noncorrected phylogenetic distance, p‐distance, and its variance were calculated across three pairs (European red deer and tule elk, European red deer and white‐tailed deer, and tule elk and white‐tailed deer) to remove regions with distance or variance <0.001, as higher phylogenetic distance between inferred sequences is more suitable for species identification. The primers for PCR amplification of HQHV regions were designed using Primer 3 (Untergasser et al., 2012). Two nuclear markers (C02 and C22) were established from these procedures.

**Table 1:** Profile of genome assembly using the next‐generation sequencing (NGS) data

Species	Accession	Reference	*N*	Total read (after QC)	Mapped read (%)
European red deer *Cervus elaphus* hippelaphus	PRJNA324173	Bana et al. (2018)	1	196,040,467	149,855,441 (76.4)
Tule elk *Cervus canadensis* nannodes	PRJNA345218	Mizzi, Lounsberry, Brown, and Sacks (2017)	4	1,584,760,432	1,363,632,036 (86.0)
White‐tailed deer Odocoileus virginianus texanus	PRJNA317745	Seabury et al. (2011)	1	2,856,626,392	1,916,901,410 (67.1)

### 2.3 DNA extraction, PCR, and Sanger sequencing

Mitochondrial and nuclear DNA were extracted from all tissue and blood samples using the standard phenol chloroform method following proteinase K treatment. Genomic DNA from antler specimens was extracted using the TBONE EX KIT (DNA Chip Research Inc.). Two previously established primers (Cb‐GS2 and Cb‐GSR2; Yamashiro, Yamashiro, Baba, Endo, & Kamada,^2010^) were used to amplify mitochondrial *cytochrome b*(Cytb). New PCR profiles were established for the novel nuclear markers. The reaction mixture (10 µl) contained 1 µl of DNA, 5.0 µl of 2× Gflex PCR Buffer, 0.2 µM of each primer, 0.2 µl of Tks Gflex DNA Polymerase (TaKaRa Bio, Otsu, Japan), and distilled water. Primers for C02 were AGCATGTAGACCCCAGACCA (forward) and GGGACCACCAAGAGACTGTG (reverse); primers for C22 were TCCGAGTTCCTAGGCTGAGA (forward) and GGCTGAAATGCTTTTTACGTCTAC (reverse). The thermocycling conditions for Cytb amplification were as follows: 94°C for 2 min; 40 cycles at 94°C for 30 s, 55°C for 30 s, and 72°C for 60 s; followed by a final extension at 72°C for 10 min. The nuclear sequences were amplified under the following conditions: 94°C for 1 min; 30 cycles at 98°C for 30 s, 62°C for 10 s, and 68°C for 30 s; followed by extension at 72°C for 3 min. The resulting amplicons were purified using the FastGene Gel/PCR Extraction Kit (NIPPON Genetics Co. Ltd.), and then sequenced using an ABI PRISM 310 DNA sequencer (Applied Biosystems, Forster City, CA, USA) with a DYEnamic ET Terminator Cycle Sequencing Kit (GE Healthcare), following manufacturer's protocol. The same primers used for PCR amplification were applied for sequencing. The samples from Osaka Prefecture, all steps of DNA extraction and sequencing were conducted twice, in two different laboratories, and by two experimenters to make the results more accurate.

### 2.4 Constructing the phylogenetic network

All inferred and directly sequenced regions were aligned using MEGA 6.06 (Tamura, Stecher, Peterson, Filipski, & Kumar, 2013) with default settings. A phylogenetic network was constructed in SplitsTree4 (Huson & Bryant, 2006). Noncorrected p‐distance was used for all three markers. Default settings in SplitsTree4 were selected for Cytb, but “average states mode” was selected for C02 and C22 regions because the nuclear sequences contained heterozygous sites. Network accuracy was estimated with 100,000 bootstraps.

### 2.5 PCR‐RFLP for genetic assessment

We used five samples—two Okinoshima deer, two control deer (Japanese and Formosan sika deer), and one Misaki Town deer samples—for the PCR‐RFLP assessment. Genotyping was performed using PCR‐RFLP. The NEBcutter V2.0 tool (http://nc2.neb.com/ NEBcutter2/; New England Biolabs Inc.) identified Rsa I (Takara Bio Inc.) as appropriate restriction enzymes for Cytb and C02, while HpyCH4IV (New England Biolabs Inc.) was identified for C22. Reaction mixtures (10 μl) were prepared following the standard protocol, with 5 μl of PCR products, restriction enzyme (0.25 μl Rsa I and 0.1 μl HpyCH4IV), buffer (0.5 μl of 10× T Buffer, 0.5 μl of 0.1% BSA, and 1 μl of 10× NEBuffer), and distilled water. Mixtures were incubated at 37°C for 3 hr before the analysis of band pattern of each region by 3% agarose gel electrophoresis (100 V, 30 min)

## 3 RESULTS

### 3.1 PCR primer establishment

Three HQHV regions were detected using the NGS data (Table 2), after excluding seven candidate HQHV regions for possessing a high number of N or heterozygous sites and two for lacking diversity. We established three nuclear DNA primer sets for the C02, C18, and C22 regions. However, based on preliminary analyses, we excluded C18 because it was not sufficiently polymorphic. Thus, we used only C02 and C22 for all subsequent analyses. Neither region contained any genes based on the cattle genomic data.

**Table 2:** Quality and genetic distance of each high‐quality and high variable (HQHV) region

Region	Position in the cattle genome	Number of N or heterozygous site			Coverage mean			Number of variant	*p*‐distance				Note
		ER	TE	WT	ER	TE	WT		ER‐TE	ER‐WT	TE‐WT	Variance	
C02	GK000002.2:109,854,351–109,855,050	2	0	0	43	56	4	5	0.01	0.042	0.04	0.0002	Primer established (C02)
C18	GK000018.2:33,116,085–33,116,784	0	0	4	42	58	4	5	0.007	0.056	0.055	0.0005	Primer established but not reported (see Results)
C22	GK000022.2:18,029,148–18,029,847	0	0	1	45	54	4	6	0.003	0.056	0.057	0.0006	Primer established (C22)

Note

ER: European red deer, TE: tule elk, WT: white‐tailed deer

Using the nuclear markers C02 and C22, we sequenced the HQHV regions from both Frm and Chb control populations. In the Frm samples, C02 yielded two (O1 and F1) diploid genotypes, whereas C22 identified one (o1). In the Chb samples, we found two diploid genotypes (J2 and J3, j1 and j2) in the C02 and C22 regions, respectively. The two control populations differed at five sites (24, 34, 224, 280, and 313 nt) in C02 (Table 3) and at three sites (64, 198, and 396 nt) in C22 (Table 4). Thus, both markers appeared suitable for distinguishing between Formosan and Japanese sika deer. A comparison of inferred sequences with the actual sequenced data revealed several gaps in C02 (at 23, 24, and 322–329 nt), but not in C22.

**Table 3:** Location of polymorphisms for each diploid genotype in the C02 region (496 bp)

Diploid genotype	Site (*n*)	Total	23	24	34	60	76	77	92	97	124	126	130
J1	Awj (15), Nwk (1) and Swk (8)	24	C	G	T	A	C	A	T	C	G	A	G
J2	Chb (2), Snn (1), Nwk (5) and Swk (7)	15	*	*	A	*	*	*	*	*	*	*	*
J3	Chb(1) and Nwk (3)	4	*	*	T/A	*	*	*	*	*	*	*	*
J4	Swk (2)	2	*	*	A	*	*	*	*	*	*	*	*
J5	Msk (1)	1	*	G/A	*	*	*	*	*	*	*	*	*
O1	Frm (1) and Oki (5)	6	*	A	*	*	*	*	*	*	*	*	*
O2	Oki (5)	5	*	A	*	*	*	*	*	*	*	*	*
O3	Oki (3)	3	*	A	*	*	*	*	*	*	*	*	*
O4	Oki (1)	1	*	A	*	*	*	*	*	*	*	*	G/A
F1	Frm (2)	2	*	A	*	*	*	*	*	*	*	*	G/A
European red deer	Inferred		–	*	*	*	*	*	*	N	*	*	*
Tule Elk	Inferred		*	–	*	*	*	*	*	A	*	*	*
White‐tailed deer	Inferred		–	*	*	G	T	G	C	*	A	T	*

Diploid genotype	Site (*n*)	Total	141	189	218	222	224	238	240	244	245	246	251	254	260	280	313
J1	Awj (15), Nwk (1) and Swk (8)	24	C	A	G	C	A	A	T	A	C	G	C	G	A	G	A
J2	Chb (2), Snn (1), Nwk (5) and Swk (7)	15	*	*	*	*	*	*	*	*	*	*	*	*	*	*	*
J3	Chb(1) and Nwk (3)	4	*	*	*	*	*	*	*	*	*	*	*	*	*	*	*
J4	Swk (2)	2	*	*	*	*	*	*	*	*	*	*	*	*	*	*	A/C
J5	Msk (1)	1	*	*	*	*	A/G	*	*	*	*	*	*	*	*	G/T	*
O1	Frm (1) and Oki (5)	6	*	*	*	*	G	*	*	*	*	*	*	*	*	T	*
O2	Oki (5)	5	*	*	*	*	G	*	*	*	*	*	*	*	*	T	*
O3	Oki (3)	3	*	*	*	*	G	*	*	*	*	*	*	*	*	T	*
O4	Oki (1)	1	*	*	*	*	G	*	*	*	*	*	*	*	*	T	*
F1	Frm (2)	2	*	*	*	*	G	*	*	*	*	*	*	*	*	T	*
European red deer	Inferred		Y	*	A	*	G	G	C	T	*	A	T	*	–	*	*
Tule Elk	Inferred		*	*	*	*	G	*	*	*	*	*	*	*	–	*	*
White‐tailed deer	Inferred		*	T	*	G	G	G	C	*	T	*	*	A	–	*	*

Diploid genotype	Site (*n*)	Total	322–329	331	338	342	361	374	395	397	422	429	439	447	463
J1	Awj (15), Nwk (1) and Swk (8)	24	–	T	A	T	T	A	A	T	T	A	A	T	T
J2	Chb (2), Snn (1), Nwk (5) and Swk (7)	15	–	*	*	*	*	*	*	*	*	*	*	*	*
J3	Chb(1) and Nwk (3)	4	–	*	*	*	*	*	*	*	*	*	*	*	*
J4	Swk (2)	2	–	*	*	*	*	*	*	*	*	*	*	*	*
J5	Msk (1)	1	–	*	*	*	*	*	*	*	*	*	*	*	*
O1	Frm (1) and Oki (5)	6	–	*	*	*	*	*	*	*	*	*	*	*	*
O2	Oki (5)	5	–	*	*	*	*	*	*	*	*	G	*	*	*
O3	Oki (3)	3	–	*	*	*	*	*	*	*	*	A/G	*	*	*
O4	Oki (1)	1	–	*	*	*	*	*	*	*	*	G	*	*	*
F1	Frm (2)	2	–	*	*	*	*	*	*	*	*	*	*	*	*
European red deer	Inferred		GATATCTA	C	G	*	*	*	*	*	*	*	–	*	*
Tule Elk	Inferred		GATATCTA	C	G	*	*	*	*	*	*	*	–	*	*
White‐tailed deer	Inferred		GATATCTA	C	*	G	A	C	T	C	C	*	–	C	C

* identical nucleotide.

**Table 4:** Location of polymorphisms for each diploid genotype in the C22 region (412 bp)

Diploid genotype	Site (*n*)	Total	6	28	32	33	55	56	64	130	141	192	197
j1	Chb (1), Snn (1), Awj (6), Nwk (9) and Swk (17)	34	T	T	C	A	G	C	C	A	T	T	C
j2	Chb (2) and Awj (9)	11	*	*	*	*	*	*	T	*	*	*	*
j3	Msk (1)	1	*	*	*	*	*	*	*	*	*	*	*
o1§	Frm (3) and Oki (9)	12	*	*	*	*	*	*	*	*	*	*	*
o2	Oki (3)	3	*	*	*	*	*	*	*	*	*	*	*
o3	Oki (1)	1	*	*	*	*	*	*	*	*	*	T/C	*
o4	Oki (1)	1	*	*	*	*	*	*	*	*	*	*	*
Tule Elk	Inferred	–	*	*	*	*	*	*	*	*	*	*	*
White‐tailed deer	Inferred	–	C	G	A	G	T	T	*	C	C	*	G

Diploid genotype	198	207	251	254	270	273	288	324	341	350	354	362	373	393	396	402	409
j1	C	A	C	C	G	T	G	G	G	A	T	A	T	C	G	C	A
j2	*	*	*	*	*	*	*	*	*	*	*	*	*	*	*	*	*
j3	C/T	*	*	*	*	*	*	*	*	*	*	*	*	*	G/A	*	*
o1§	T	*	*	*	*	*	*	*	*	*	*	*	*	*	A	*	*
o2	C/T	*	*	*	*	T/G	*	*	*	*	*	*	*	*	G/A	C/T	*
o3	C/T	*	*	*	*	T/G	*	*	*	*	*	*	*	*	G/A	C/T	*
o4	C/T	*	*	*	*	*	*	G/T	*	*	*	*	*	*	G/A	C/T	*
Tule Elk	T	*	*	*	*	*	*	*	A	*	*	*	*	*	A	*	*
White‐tailed deer	C	G	T	G	C	*	C	*	*	G	C	G	C	T	*	T	G

§ Same as inferred sequence of European red deer.

* identical nucleotide.

### 3.2 Evaluation of the Honshu and Awajishima Island populations

**Figure 3:**
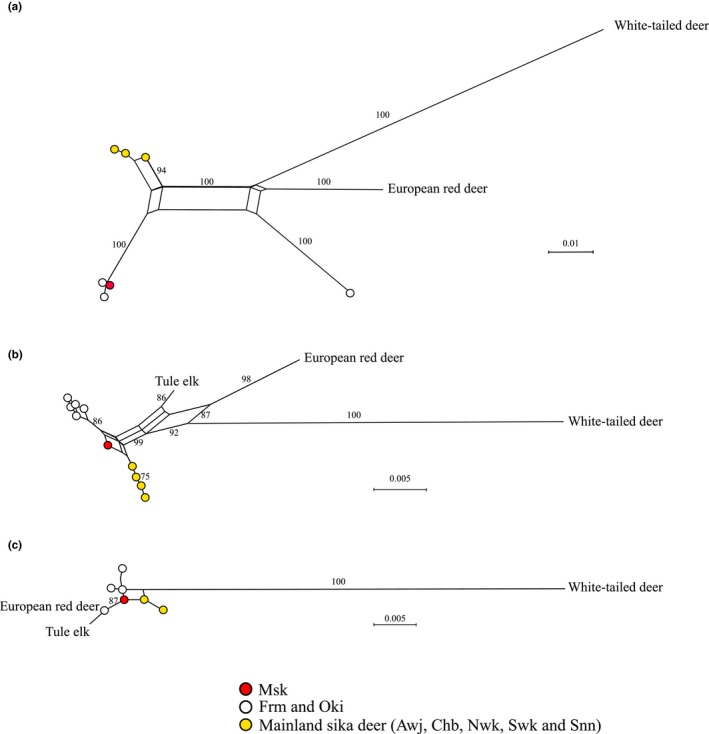
Neighbor‐Net phylogenetic trees for mitochondrial *cytochrome b* (a), C02 (b), and C22 (c). The values indicate bootstraps with 100,000 replications (only those exceeding 75 are shown)

The Misaki Town specimen exhibited a Cytb haplotype identical to the haplotype in Formosan sika deer, also previously found in the Okinoshima population (Matsumoto et al., 2015). Our Neighbor‐Net phylogenetic analysis classified the remaining 42 deer as Japanese sika; thus, they should have haplotypes similar to those of the Northern Japanese and Chb populations (Figure 3a).

We found five diploid genotypes (J1–J5) for the C02 region in the Misaki, Sennan, North Wakayama, South Wakayama, and Awajishima populations (Figure 3b, Table 3). The Awajishima population exhibited only one diploid genotype (J1). The genotypes J2 and J3 were present in Chb, as well as in the North and South Wakayama deer, whereas two South Wakayama individuals possessed J4. The Misaki individual was the only one to exhibit genotype J5. Furthermore, the Okinoshima population had four distinct C02 alleles (O1, O2, O3, and O4).

The Honshu and Awajishima populations exhibited three diploid genotypes (j1–3) in the C22 region (Figure 3c, Table 4). The most common genotype was j1 (*n* = 34), differing from j2 by only one substitution at 64 nt. Similar to C02, the Okinoshima population contained four diploid genotypes in the C22 region. The most common allele in the Okinoshima population was o1; its sequence was identical to that of Formosan sika deer and the inferred sequence of European elk. The phylogenetic analysis revealed that the Okinoshima population has two distinct nodes in the C22 region (Figure 3c).

The Misaki sample is heterozygous at C02 (J5) and C22 (j3). These two diploid genotypes were identical to the homozygous sequences (J1 and O1, j1 and o1) found in other populations. The phylogenetic analysis using both C02 (Figure 3b) and C22 (Figure 3c) indicated that the Misaki sample was between the Okinoshima and Japanese sika deer clades.

### 3.3 PCR‐RFLP

**Figure 4:**
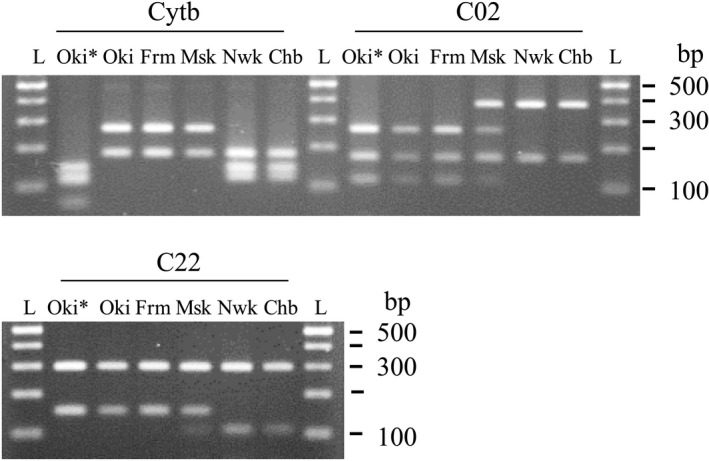
Electrophoresis of polymerase chain reaction–restriction fragment length polymorphism (PCR‐RFLP) amplicons from the Okinoshima population (*sambar‐type *cytochrome b* sequence), Japanese sika deer, Formosan sika deer, and a hybrid deer (Msk). L: 100‐bp ladder

The PCR‐RFLP analysis of the five samples confirmed the sequencing results (Figure 4). In the Okinoshima population, the sambar‐type Cytb sequence differed from the Formosan sika‐type sequences. However, the Okinoshima‐origin fragment patterns were identical to Formosan sika and Misaki samples. The fragment patterns of both C02 and C22 differed between the foreign populations (Okinoshima, Formosan sika) and the native Japanese population (North Wakayama, Chb). Furthermore, the Misaki fragment patterns were heterozygous for C02 and C22, with one allele from the foreign deer and the other from Japanese deer.

## 4 DISCUSSION

### 4.1 Introgressive hybridization

Our analyses using novel nuclear DNA markers (C02 and C22) and an existing mitochondrial marker supported an apparent phylogenetic distinction between Formosan sika and Japanese sika deer, as demonstrated previously (Matsumoto et al., 2015; Randi, Mucci, Pierpaoli, & Douzery, 2001). Notably, the Misaki Town sample possessed mitochondrial DNA from Formosan sika deer. Furthermore, all the SNP sites of the two nuclear sequences were heterozygous, with an allele from the native Japanese sika deer and an allele from the hybrid Okinoshima population. Our analysis of the alpha‐lactalbumin gene (Matsumoto et al. *unpublished data*) showed a similar heterozygosity. To the best of our knowledge, purebred Formosan sika deer have not been introduced or found around Misaki. Therefore, the Misaki individual is probably a first‐generation hybrid between Okinoshima deer and native Japanese sika deer.

Male deer disperse more frequently than female deer (Clutton‐Brock, Guinness, & Albon, 1982; Ito & Takatsuki, 2009); therefore, it is not surprising that only males have been observed on Jinoshima Island, located between Okinoshima Island and Honshu (Wild Management Organization, 2017). However, the fact that our Misaki sample has a maternal lineage originating from Okinoshima indicates that does also swam to Honshu at some point and mated with Japanese sika males. In contrast, Japanese sika deer have not managed to migrate from Honshu to Okinoshima Island, based on the sequence identified in the Okinoshima population so far (Matsumoto et al., 2015).

Our data suggest introgressive hybridization in the Okinoshima population. First, at least three Cervidae species (Formosan sika deer, Formosan sambar, and red deer) may have been mated for antler production in Taiwanese deer facilities (Matsumoto et al., 2015). Second, members of this Okinoshima population may have mated with the native Japanese sika deer on Honshu resulting in another hybridization event.

### 4.2 Evaluation of Wakayama and Awajishima Island populations

All three DNA sequences of the 42 deer from three Japanese populations (Awajishima, North Wakayama, and South Wakayama) were identical to typical Japanese sika deer sequence and were in the same clade as the Chb population. Thus, we found no evidence of introgression in the Wakayama Prefecture or Awajishima Island.

There are two limitations for the detection of introgressive hybridization in this study. First, the detection of other hybridization events may have been impaired by the limited sample sizes. Further genetic assessments using larger sample sizes are therefore needed. Second, a low number of markers were used and, in general, this may also hinder the detection of the hybridization (Dupuis et al., 2012). Nevertheless, low deer density in Misaki Town and surroundings and the fact that no hybridization without Misaki sample was detected indicate that the hybridization event between Okinoshima population and native Japanese sika deer may be limited. Our genetic assessment using the three markers, which can be used to diagnose hybridizations at least in F1 generation hybrids, will maximize the detection of hybridization in this stage. However, further crosses between these populations may reduce the detection rate of our method. In this case, more markers resulting from NGS data will be needed to diagnose subspecies and determine hybridization rate.

### 4.3 Conservation of Japanese sika deer

Japanese sika deer is an endemic species in Japan and is highly divergent in terms of phenotypic (Terada et al., 2012) and genetic features (Nagata et al., 1999). A phylogenetic study using mitochondrial DNA suggested that 4.9–6.7 million years may have passed since the divergence of Japanese and other *C. nippon* subspecies including Formosan sika deer (Randi et al., 2001). In addition to the phylogenetic difference, the “Nara deer,” a national treasure in Nara Prefecture, Kinki District, has been protected by law since the 13th century, and the importance of the deer involves historical, cultural, and also tourism perspectives (Torii & Tatsuzawa, 2009). Therefore, further hybridization should be avoided and the original genetic integrity of the Japanese sika deer population should be conserved.

### 4.4 Management of the Okinoshima population

Recently, a re‐introduction program has been implemented for the Formosan sika deer, and the population size has exceeded 1000 individuals in Kenting National Park, Taiwan (Pei & Liang, 2015). However, the genetic diversity of the re‐introduced population is low because of the limited number of founders; therefore, outcrossing is necessary (Pei & Liang, 2015). The results of a previous study (Matsumoto et al., 2015) and of the present one indicate that many genotypes are shared between Formosan sika deer and the Okinoshima population, suggesting that the major genetic component of the latter could be from the former. Therefore, the Okinoshima population could be used as a genetic resource for the re‐introduced population in Taiwan, although further genetic assessments, such as whole genome sequencing of both Okinoshima and Formosan sika deer, are required.

Limited management efforts have been made to prevent introgression in the Okinoshima population. Two management measures—complete eradication of the hybrid population and the establishment of fences around Okinoshima Island—have been proposed (Wild Management Organization, 2017). For the Okinoshima population, we suggest that fencing is preferable to avoid dispersal, considering this populations' potential as a genetic resource for the re‐introduced population of Formosan sika deer in Taiwan.

### 4.5 Applying NGS data for conservation genetics

Researchers are increasingly using publicly available NGS data in conservation genetics (Allendorf, Hohenlohe, & Luikart, 2010). In the present study, we successfully employed NGS data to establish novel genetic markers for species identification and determining hybridization. The use of NGS data to develop genetic markers offers several advantages. First, given the inclusion of genome‐wide polymorphisms in such data, multiple copies of a single genetic region in the genome can be detected. Such duplicated regions will negatively influence phylogenetic analyses; therefore, the use of NGS data allows the exclusion of these regions and the selection of single‐copy regions instead (Naumann et al., 2011). In the present study, we searched for and sequenced C02 and C22, both single‐copy regions in the Cervidae genome. The use of genomic data in conservation and phylogenetic studies also offers the potential for detecting a genetic region correlated with adaptive phenotypes (Ouborg, Pertoldi, Loeschcke, Bijlsma, & Hedrick,^2010^ ). For example, a genomic approach in polar bears enabled successful identification of genetic regions under strong selection and linked to reorganization of the cardio‐vascular system (Liu et al., 2014). We recommend NGS data‐based primer establishment coupled with fine mapping to uncover candidate regions related to adaptive phenotypes as such discoveries can greatly benefit conservation efforts.

Mitochondrial data revealed that European red deer, tule elk, and Japanese sika deer are closely related (Ludt, Schroeder, Rottmann, & Kuehn, 2004). In support of this previous finding, we identified a shared o1 diploid genotype of C22 between Frm and European red deer. However, we also found alignment gaps at 23, 24, and 322–329 nt in the C02 sequence. These gaps probably result from mapping errors, a major limitation of NGS‐based primer establishment that should be taken into account. Therefore, future studies should obtain actual tissue samples of European red deer, tule elk, and white‐tailed deer to directly sequence the C02 region, thereby allowing a more precise estimation of phylogenetic relationships.

### 4.6 Conclusion

The present study uncovered introgressive hybridization among Cervidae members. Currently, introgression between the Okinoshima population and native Japanese sika deer is likely rare, because we found only one hybrid individual in Honshu. However, our data suggest that at least one Okinoshima doe swam to Honshu. Moreover, in Misaki Town and surroundings, we found little evidence of sika deer existence, suggesting a low native population density. The Okinoshima deer are also hybrids with at least three parent species, that is, the introgression of their genetic material into a small Japanese sika population could have an outsized effect. However, other hybridization events may not have been detected because of the limited sample size and markers used in the present study. To have a better understanding of introgression in Japanese deer populations, we strongly recommend genetic assessment on a large scale, covering more individuals and expanding across North Kinki district. The PCR‐RFLP procedure we developed can accelerate such large‐scale assessments. We also strongly recommend the establishment of fence around Okinoshima Island, to prevent further degradation of genetic integrity among the native deer population of Honshu (Wild Management Organization, 2017).

## CONFLICT OF INTEREST

None Declared.

## AUTHOR CONTRIBUTIONS

Y.M. and H.B.T designed research, Y.M., T.T., R.K, A.Y., A.T. performed research, Y.M., T.T., A.T. contributed with the analytical tools and analyzed data, and all authors wrote the paper.

## Data Availability

Sequences were deposited in GenBank (DDBJ/EMBL) under the accession numbers LC387324‐LC387325 (Cytb), LC386632‐41(C02), and LC387317‐23 (C22).
